# Isolation, characterization, evaluation of pathogenicity, and immunomodulation through interferon production of duck adenovirus type-3 (DAdV-3)

**DOI:** 10.1016/j.psj.2023.103411

**Published:** 2024-01-04

**Authors:** Yang Tan, Muhammad Akmal Raheem, Muhammad Ajwad Rahim, Huang Xin, Yuhang Zhou, Xuerui Hu, Yin Dai, Farid Shokry Ataya, Fangfang Chen

**Affiliations:** ⁎ Key Laboratory of Veterinary Pathobiology and Disease Control, College of Animal Science and Technology, Anhui Agricultural University, Hefei 230036, Anhui, PR China; † Institute of Biopharmaceutical and Health Engineering, Tsinghua Shenzhen International Graduate School, Tsinghua University, Shenzhen 518055, PR China; ‡ Tsinghua- Berkeley Shenzhen Institute, Tsinghua Shenzhen International Graduate School, Tsinghua University, Shenzhen 518055, PR China; § Anhui Academy of Agricultural Sciences, Hefei 230036, Anhui, PR China; # Department of Biochemistry, College of Science, King Saud University, Riyadh 11451, Saudi Arabia

**Keywords:** DAdV-3, separation, pathogenicity, interferon, cytokine

## Abstract

Duck adenovirus type-3 (**DAdV-3**) is a poorly characterized duck virus. A comprehensive analysis of the DAdV-3 pathogenicity and host immune response could be a valuable addition. Herein, DAdV-3 was isolated from Muscovy duck and virus-specific genes were confirmed by polymerase chain reaction (**PCR**). The obtained gene fragments were sequenced and compared with the reference sequence. Results confirmed that the clinically isolated virus was DAdV-3, named as HF-AN-2020. To evaluate DAdV-3 host immune response, the expression levels of MDA5, STING, IRF7, MAVS, and NF-κB, and inflammatory cytokines (IFN-β, IFN-γ, and IL-1β) were determined by quantitative reverse transcriptase PCR (**qRT-PCR**). The expression levels of IFN-β and IFN-γ were 32.6- and 28.6-fold, respectively, higher (*P* < 0.01) than the control group. It was found that the upregulation of STING and NF-κB pathways was directly involved in the regulation of inflammatory cytokines (IFN-β, IFN-γ, and IL-1β). Furthermore, the gene regulation pathways consecutively upregulated the expression levels of MDA5, STING, IRF7, MAVS, and NF-κB up to 31.6, 10.5, 31.4, 2.2, and 2.6-fold, respectively, higher (*P* < 0.01) than the control group. The TCID_50_ of DAdV-3 for Muscovy duck and chicken was 10^−3.24^/0.1 mL with 0% mortality, indicating low pathogenicity in both Muscovy ducks and chickens, but DAdV-3 can induce higher expression of interferons. Genome analysis showed mutations in 4 amino acids located in ORF19B (Ser to Thr), ORF66 (Leu to Phe, Ile to Leu), and ORF67 (Gly to stop codon). This study provides essential and basic information for further research on the mechanism of the cellular immune responses against adenoviruses.

## INTRODUCTION

According to the International Committee on Taxonomy of Viruses (**ICTV**), the family *Adenoviridae* contains 6 distinct genera (*Aviadenovirus, Mastadenovirus, Atadenovirus, Siadenovirus, Ichtadenovirus*, and *Testadenovirus*) (https://talk.ictvonline.org/) (Benk\Ho et al., 2022). The family *Adenoviridae* consists of several serotypes which infect many vertebrates including mammals and birds. Adenoviruses isolated from poultry are generally classified into the genus Aviadenovirus (Marek et al., 2014). Aviadenoviruses are nonenveloped, icosahedral, and double-stranded DNA, which can infect many birds such as ducks, chickens, fowl, geese, pigeons, falcons, turkeys, and psittacine species (Davison et al., 2003). Although both DAdV-2 and DAdV-3 were isolated from Muscovy ducks, they have different viral genome structures and pathogenic characteristics. The genome of DAdV-3 contains 3 main surface structural proteins including; fiber, hexon, and penton. Like the serotype 4 fowl adenovirus (**FAdV-4**), DAdV-3 also has 2 fiber proteins (fiber-1 and fiber-2). Previous studies have shown that the fiber proteins are related to virus neutralization, so they are ideal for the detection of viruses and vaccine development (Yin et al., 2019; Shao et al., 2022; Lin et al., 2023) The amino acid sequences of hexon proteins are different in each strain which is important for virus classification (Marek et al., 2010). Penton, as a bond between hexon and fiber, plays an important role in the stability of viral capsid and the attachment of invading virion (Gao et al., 2019).

Several molecular methods such as polymerase chain reaction (**PCR**), real-time PCR, high resolution melting (**HRM**) curve analysis, and loop-mediated isothermal amplification (**LAMP**) have been used for the detection of aviadenoviruses based on the major capsid protein (hexon) or the DNA-dependent DNA polymerase (Kaján et al., 2011; Xie et al., 2011; Günes et al., 2012). DAdV-3 (GD-CH-2014) was first discovered in China in 2014. The ducks infected with DAdV-3 were characterized by swelling, bleeding, and hemorrhages of the liver and kidneys, with a morbidity rate of 40 to 55% and a mortality rate of 35 to 43%, which caused huge economic losses to the duck industry (Zhang et al., 2016; Yin et al., 2022). However low virulence strains of DAdV-3 have not been reported so far.

In this study, DAdV-3 was isolated and identified from the Muscovy duck farm, and its pathogenicity to Muscovy ducks and chickens was analyzed. The possible immune mechanism was explored to pave for further research on its prevention and control.

## MATERIALS AND METHODS

### Animals and Ethics Statement

Day-old specific-pathogen-free (**SPF**) chick and chick embryos were purchased from Zhejiang Lihua Agricultural Co., Ltd. (Anhui, China). Muscovy ducks were procured from Yongqiang Agricultural Co., Ltd. (Anhui, China). These birds were kept in a healthy and controlled environment for 30 d. The temperature was maintained according to the age and behavior of the birds (28°C–33°C). All birds were kept and handled according to the Institutional Animal Care and Use Committee (**IACUS**) guidelines by the School of Animal Science and Technology, Anhui Agricultural University, Hefei, China (AHAU 2020-010).

### DAdV-3 (HF-AH-2020) Isolation

The liver tissues of a 30-day-old DAdV-3 infected Muscovy duck were collected from the Avian Disease Diagnostic Center of Anhui Agricultural University, Hefei, China, and used as a DAdV-3 source for this study. The liver was cut into pieces and grounded into a homogenate mixture under sterile conditions. Subsequently, Hank's solution (Beyotime, Shanghai, China) was added to this homogenate mixture to make a 10% suspension. After this suspension was frozen and thawed 3 times, then centrifuged at 12,000 rpm for 30 min at 4°C. The supernatant was filtered through a 0.22 μm filter and stored at −80°C for further use.

The supernatant was inoculated with the dose rate of 0.15 mL/embryo into the allantoic cavity of 8-day-old SPF chicken embryos. The inoculated embryos were incubated at 37°C with a relative humidity of 55%. The embryos that died within 2 d were discarded, whereas those that died between 9 and 10 d were stored for the collection of DAdV-3.

### DAdV-3 (HF-AH-2020) Nucleic Acid Extraction and Phylogenetic Analysis

The total RNA and DNA were extracted by viral RNA/DNA purification kit (Tiangen, Beijing, China) according to the manufacturer's instructions. RNA was reverse-transcribed into cDNA according to manufacturer instruction (Tiangen, Beijing, China), and used as a PCR template. The *hexon* gene of adenovirus was amplified by a self-designed primer: Hexon F: ATGGCCGCTCTGACCCCTGA and Hexon R: ATTCAGCCTTAGCTACTTTC. In addition to DAdV-3, the same samples were tested for other viruses such as; duck stellate virus (**DAstV**), duck Tembusu virus (**DTMUV**), avian pulmonary virus (**AMPV**), avian influenza viruses H5 and H7 (**AIV-H5** and **AIV-H7**), and duck circovirus (**DuCV**). Optimized primers from other studies were also used in our experiments (Liu et al., 2016; Weiguo et al., 2016; Yu et al., 2019; Huang et al., 2020). The viral nucleic acid amplification and identification was done by PCR (Günes et al., 2012; Pan et al., 2017), and amplified DNA fragments were cloned into a pMD18-T vector (Dongsheng, Guangzhou, China) for sequencing (Huada, Shanghai, China). The obtained *hexon* gene sequences were aligned for gene analysis. Furthermore, 26 avian adenovirus strains; FAdV-A, B, C, D, E, and DAdV were selected for the phylogenetic analysis of hexon protein. The amino acid sequences were aligned by using BioEdit v7.2.5. The best amino acid substitution models were MEGA v6.0. and DNA star, used for evolutionary genetic analysis of the target gene using the maximum likelihood (**ML**) method, to obtain the phylogenetic tree of the isolated strain (DAdV-3).

### Cytopathic Effect (CPE) of DAdV-3 (HF-AH-2020)

Chicken embryonic kidney (**CEK**) cells were cultured according to previously reported methods (Li et al., 2018, 2021). CEK cells were inoculated in 6 well plates with a ratio of 1 × 10^7^/well overnight. After overnight incubation, the supernatant of these cultured cells was discarded, 500 µL serum-free RPMI medium (Hyclone, South Logan, Utah) modified with 10 µL allantoic fluid of Muscovy duck, and 30 µL allantoic fluid of chicken embryo was added to each well, then incubated for 1 to 2 h, and last 2 wells were used as negative control (only PBS). Subsequently, the supernatant was discarded, RPMI Medium Modified with 1% FBS was added, and changes in the cells were observed daily.

### RT-PCR-Based Organ-Specific Detection of DAdV-3 (HF-AH-2020)

To analyze the distribution of the virus in different organs (heart, liver, spleen, kidney, brain, forestomach, glandular stomach, and intestine) of chicken embryos, 3 embryos within an hour of death were used to collect the 50 mg tissue sample taken from each above-mentioned organs for DNA extraction. The primers for DAdV-3 were designed for the highly conserved region of the DAdV-3 52K gene. The primers sequences were followed as 52k-F: 5′-TGTGGAAATCGGGGTGTATC-3′, 52k-R: 5′-GTCAAACCGAACATGTAGTCTG-3′. The plasmid pMD18-T-52K was also designed. The quantification of DAdV-3 through real-time PCR (**RT-PCR**) was done by using AceQ qPCR SYBR Green Master Mix (Vazyme, China). The standard curves were obtained from 3 independent experiments each performed in duplicates by using 10-fold serial dilutions of plasmid (pMD18-T-52 K) and its identification was done by RT-PCR. The number of viral copies per gram was calculated by comparing the values of the threshold cycle (**CT**) with standard curve values. Each reaction was performed in triplicate, to get mean ± standard deviation (**SD**). The calculated values of viral load were expressed as the number of viral copies per gram of tissues (No. VC/g tissue).

### Amplification and Structural Analysis of DAdV-3 (HF-AH-2020) Whole Genome

The viral DNA was extracted from the liver of DAdV-3 infected Muscovy duck by using a DNA extraction kit (Anheal, Beijing, China). Genomic DNA was quantified by using a TBS-380 fluorometer (Turner BioSystems Inc., Sunnyvale, CA). The high-quality DNA samples (OD260/280 = 1.8–2.0, >6 ug) were used to construct a library of DNA fragments. The complete viral genome was sequenced by Illumina Hiseq combined with third-generation sequencing technology. Sequencing and genome assembly was performed by Shanghai Biozeron Biotechnology Co, Ltd. (Shanghai, China). The genome sequence was submitted to NCBI, and the nucleotide sequence was compared to the NCBI GenBank database, using the BLAST Search Tool (**BLASTn**). The amino acid sequences of the putative proteins were also compared to proteins, present in the NCBI GenBank database by using the protein (**BLASTp**). Moreover, Snapgene 4.2.4 software was also used to compare and analyze the sequences of amino acids isolated with a virulent strain of DAdV-3 (GD-CH-2014).

### DAdV-3 (HF-AN-2020) Virus Titration

Chicken hepatocellular carcinoma (LMH) cells were cultured in a culture medium containing 10% FBS (GE, Utah) and DMEM/F12 (Biosharp, Beijing, China). This media was spread in 96-well culture plates at the ratio of 1 × 10^4^ cells/100 μL. These plates were incubated overnight with 5% CO_2_ at 37°C. The virus was diluted 10-fold (1/10) with PBS, followed by 8 gradients of 10^1^, 10^2^, 10^3^, 10^4^, 10^5^, 10^6^, 10^7^, and 10^8^ in the total volume of 100 μL/well, and the last 2 wells were kept as negative control (only PBS). After incubation at 37℃ for 2 h, the culture media of all wells were replaced with a 0.1 mL culture medium containing 1% FBS for incubation for up to 9 d. During culture, the characteristics of infected cells were; round, aggregation, enhanced refractive index, and typical beads on a string. The period of infection and death of the cells was recorded as 50% Tissue Culture Infective Dose (**TCID_50_**) according to the Reed-Muench formula (Matumoto, 1949).

### Isolation of Bacteria and Identification of Virulence Island

In order to verify whether the isolated HF-AN-2020 strain was coinfected with bacteria or not, bacteria were isolated and identified from the liver of a Muscovy duck at the same time. The liver of the Muscovy duck was dissected under aseptic conditions and cultured in a TSB medium (BD, Maryland) at 37°C with 5% CO_2_ for 48 h. After this, selecting 1 colony for Gram staining and PCR identification of bacterial 16sRNA was done. The preliminarily identified *E. coli* was amplified and recognized for the virulence island gene. The identification methods of bacteria and their virulence was performed according to the previously used method (Wang et al., 2015).

### Animal Trials

Fourteen-day-old SPF chickens and Muscovy ducks were randomly divided into 8 groups, 15 in each group. The 0.25 mL virus (HF-AN-2020) was introduced by intramuscular (**IM**) injection in the thigh muscles of chickens and Muscovy ducks with a dose rate of 10^−3.24^ TCID_50_/0.1 mL for the positive control groups whereas normal saline was injected in the negative control group with the same dose rate (0.25 mL of PBS). All birds were kept according to the standard protocols of IACUS and monitored for 12 d of postinfection. After fourth day of infection, cloacal swabs were collected from 3 chickens and 3 ducks in each group, and livers were extracted for virus detection after the euthanization of the birds. Meanwhile, the liver and kidney samples were collected from each group and sent to Servicebio Biotechnology Co, Ltd. (Wuhan, China) for histopathological analysis. On the 12th day of postinfection, 0.5 mL blood was drawn from the wing veins of both birds (chickens and ducks) via a syringe and serum was separated for the detection of antibody titers.

### Enzyme-Linked Immunosorbent Assay

The indirect enzyme-linked immunosorbent assay (**ELISA**) method was used to determine the DAdV-3-specific antibody titers in the serum of all experimental birds. Twenty mL of allantoic fluid was collected from infected (DAdV-3) chicken embryos. For the isolation of DAdV-3, the infected allantoic fluid was centrifuged at 28,000 rpm for 4.5 h to obtain 1 mL of concentrated allantoic fluid. The protein concentration was detected with the BCA protein concentration assay kit (Beyotime, Shanghai, China). Hundred µL of concentrated DAdV-3 (HF-AH-2020) (1 µg/µL) was placed into each well of the ELISA plates and incubated overnight at 4 ℃. After incubation, the plates were washed 3 times with PBS containing 0.05% Tween-20 (**PBST**) and then incubated with 5% FBS (Beyotime, Shanghai, China) for 1 h at 37°C. After 3 washes, chicken or duck serum samples were diluted with a ratio of 1:20,000 and again incubated at 37°C for 1 h. The following incubated samples were again washed 3 times with PBST and incubated for 1 h at 37°C with HRP-conjugated goat Anti-Bird IgY (Abcam) via dilution rate of 1:5,000. After washing, 100 μL tetramethylbenzidine substrate (Beyotime, Shanghai, China) was added to each well, and the plates were incubated in the dark for 10 min. The enzymatic reaction was quenched by hydrofluoric acid, and the optical density (**OD**) was determined at 450 nm. Meanwhile, The serum of negative chickens or ducks was negative control. Sample value was calculated as a ratio using the formula: value = ODsample − ODneg. Each sample was analyzed in triplicate.

### Detection of Interferon β and γ Genes Expression by qRT-PCR

To investigate the reason for the low pathogenicity of DAdV-3 (HF-AH-2020), we detected the expression of interferons and related genes after 72 h of infection with CEK cells. The experiment of CEK cells infected with DAdV-3 (HF-AH-2020) was repeated 3 times. The reference sequence of cytokines (IFN-β, IFN-γ, and IL-1β), signaling pathways regulating molecules NF-κB and stimulator of interferon genes (**STING**), membrane-related receptor (**MDA5**), interferon regulatory factor-7 (**IRF7**) and signal protein (MAVS) genes were used for primer designing via GenBank. The names of the genes, primer sequences, amplified fragment size, and login ID numbers have been taken from previous studies (Li et al., 2021).

Cytokines (IFN-β, IFN-γ, and IL-1β), signaling pathways regulating molecules (NF-kB, STING), membrane-related receptor (MDA5), signal protein IRF7 and MAVS were detected by using cDNA as a template through real-time reverse transcription-PCR (**qRT-PCR**). At the same time, FAdV-4 infected cells were used as a control group, and relative expression was calculated by the 2^−ΔΔCT^ method.

### Statistical Analysis

The statistical analysis was performed by SPSS 16.0 (SPSS Inc., Chicago, IL). The results of all experiments were analyzed by *t* test, followed by Tukey's honestly significant differences (**HSD**) for posthoc testing to compare the significance (*P*) between the means of different groups. The differences were considered statistically significant at *P* < 0.01.

## RESULTS

### Identification and Phylogenetic Analysis of DAdV-3 (HF-AH-2020)

**Figure 1:**
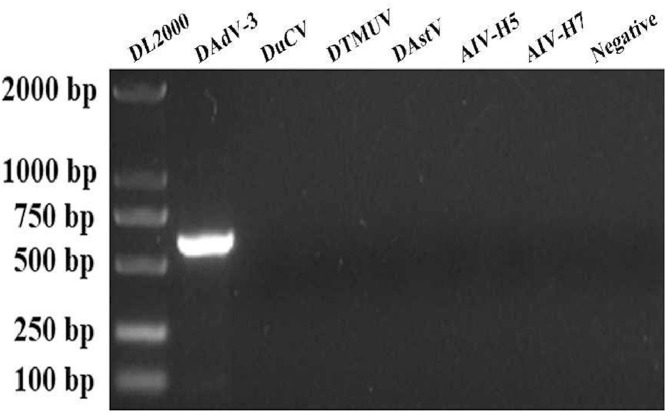
Detection of the DAdV-3 target sequence 600 bp by PCR analysis is shown in lane 2. Lanes 3 to 7 DuCV, DTMUV, DAstV, AIV-H5, and AIV-H7 were showing no interference with the target sequence, Lane 8 is negative control (PBS).

**Figure 2:**
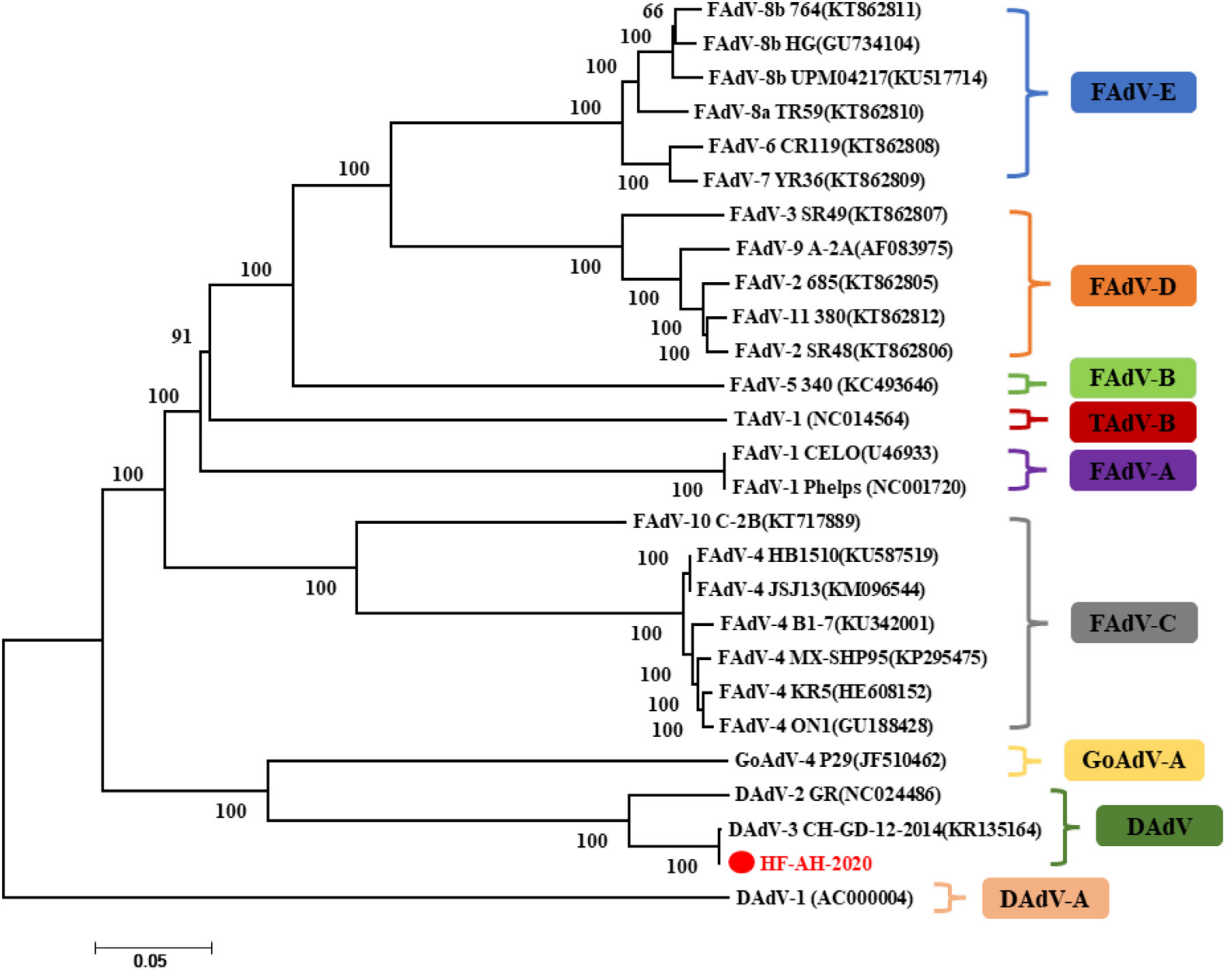
Phylogenetic analysis of DAdV-3 (HF-AH-2020) hexon gene with different serotypes of adenoviruses. The evolutionary history was inferred by using the maximum likelihood method based on the Tamura-Nei model. The percentages of replicate trees in which the associated taxa clustered together in the bootstrap test (1,000 replicates) are shown below the branches. Evolutionary analyses were conducted in MEGA6.0 software.

The genomic DNA and cDNA were extracted from DAdV-3 suspected liver of Muscovy duck, and used as a PCR template to determine DAdV-3-specific gene fragment (600 bp). Figure 1 shows the successful detection of the DAdV-3 target sequence (HF-AH-2020). Another hand, when cDNA was used as a template, then as a result no band was found of any other kind of viruses such as the duck star virus (DAstV), duck Tambusu virus (DTMUV), H5 avian influenza virus (AIV-H5) and H9 avian influenza virus (**AIV-H9**) (Figure 1) indicating the sole presence of DAdV-3 in the sample. After comparison with other sequences in NCBI, it was found that the amplified *hexon* gene sequence was 100% homologous to the reported DAdV-3 (CH-GD-2014) strain, which was on the same branch as DAdV-2 and belonged to duck aviadenovirus B (**DAdV-B**), but it has no resemblance with other adenoviruses (Figure 2). The homology of DAdV-3 with DAdV-1 (KF286430) and FAdV-4 (KU587519) was 52.8 and 65.2%, respectively. Although different DAdV has the same capsid proteins (hexon, fiber) but the homology is quite different. As shown in Table 1, the amino acid homology of DAdV-3′ hexon, fiber-1, or fiber-2 with DAdV-2 was 86.9, 15.3, or 28.5%, respectively. Similarly, the homology of DAdV-3′ hexon, fiber-1, and fiber-2 with DAdV-4 was 72.3, 17.1, and 23.2%. The homology of DAdV-3′ hexon, fiber1, and fiber2 with goose adenovirus 4 (GoAdV-4, JF510462) was 82.4, 25.8, and 37.1%. This shows that DAdV-3 has little homology as with other viruses, especially for fiber protein (Table 1).

**Table 1:** Homology comparison of DAdV-3 genome with different DAdVs domains.

DAdV-3 domains	DAdV-2	DAdV-4	GoAdV-4
Genome	92.3%	57.2%	57.4%
Hexon	86.9%	72.3%	82.4%
Fiber-1	15.3%	17.1%	25.8%
Fiber-2	28.5%	23.2%	37.1%

### Whole Genome Sequence Analysis of DAdV-3 (HF-AH-2020)

**Figure 3:**
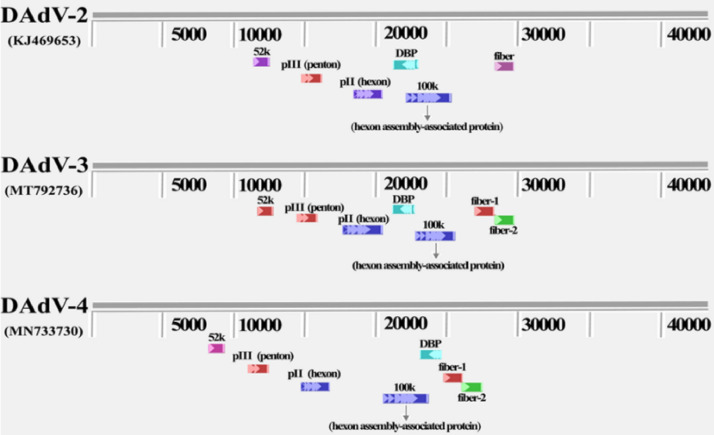
The locations of major structural proteins (hexon, fiber, penton) in DAdV-2, DAdV-3, and DAdV-4 genomes were different. All DAdVs have hexon, fiber, penton, 52K, 100K, and DBP (DNA binding protein), but their position and homology were different in different DAdVs, especially among DAdV-2 and DAdV-3.

The complete genome sequence of a DAdV-3 was obtained by sequencing the whole genome, and complete amplified genome DAdV-3 (HF-AH-2020) sequencing data were submitted to the GenBank, under the accession number MT792736. The whole genome sequencing analysis showed that DAdV-3 nucleotides were homologous to DAdV-2 and DAdV-4 with 92.3 and 57.2%, respectively. As shown in Figure 3, the main structural proteins of DAdVs were include 52K, pII (hexon), pIII (penton), DBP (DNA binding protein), 100K, and fiber. DAdV-3 (MT792736) and DAdV-4 (MN733730) had 2 fibers (fiber-1 and fiber-2), but the locations of these both fibers (proteins) were different in different viral genomes, while DAdV-2 (KJ469653) has only 1 fiber (Figure 3). Snapegne analysis shows that the main amino acid changes in HF-AH-2020 and GD-CH-2014 were ORF19B(Ser to Thr), ORF66(Leu to Phe, Ile to Leu), and ORF67(Gly to stop codon), and there were 1 to 2 amino acid mutations in each of these ORFs, among which the mutation of ORF67 lead to early termination of ORF67 protein translation.

### Distribution of DAdV-3 (HF-AN-2020) in Embryonic Stage

**Figure 4:**
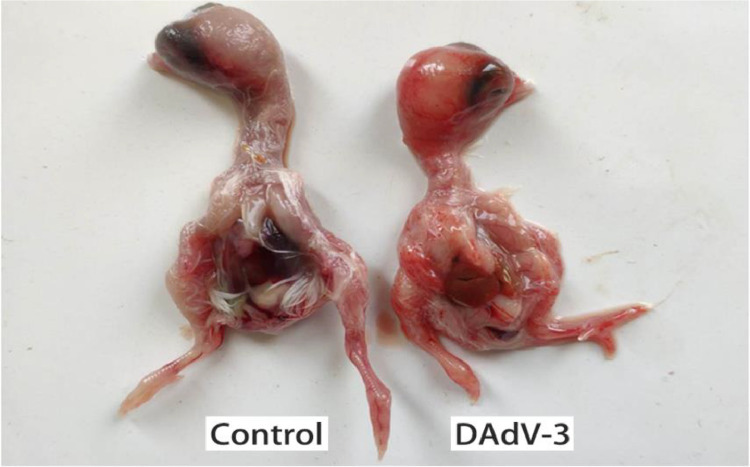
DAdV-3 infected chicken embryos were showing short and stunted growth, the whole-body surface was covered with a large number of petechial hemorrhages, and the liver showed punctate necrosis, while the control negative group (PBS) was normal.

**Figure 5:**
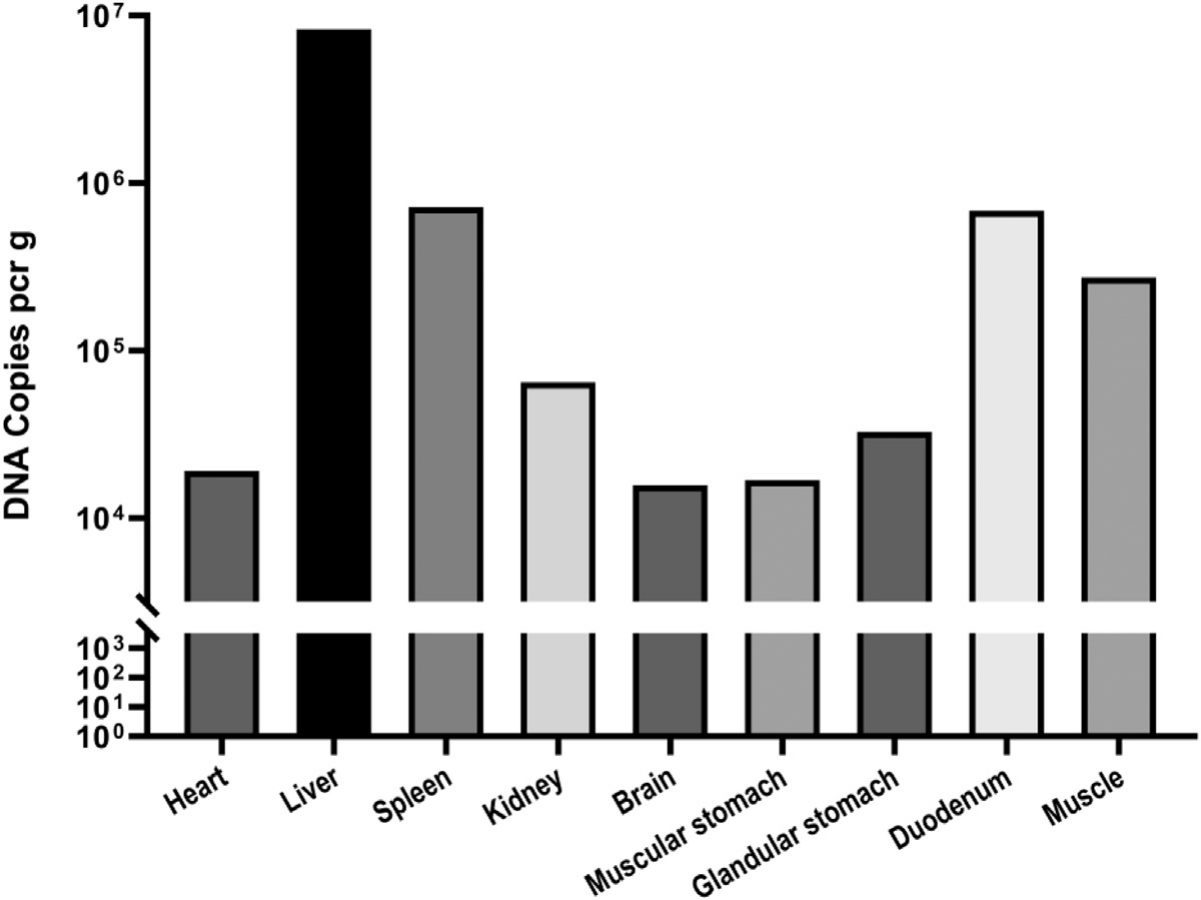
The DAdV-3 (HF-AN-2020) viral load was higher in the liver, whereas the other organs' viral load sequence was the spleen, duodenum, kidney, glandular stomach, heart, muscle, muscular stomach, and brain.

SPF chicken embryos were died within 9 to 10 d after DAdV-3 inoculation. The DAdV-3 infected chicken embryos were short and stunted, and the whole-body surface was covered with a large number of petechial hemorrhages. Further, punctate necrosis was observed on the surface of the liver, while it was a normal texture in the control group (Figure 4). Three embryos within an hour of death were used to collect the tissues (50 mg) from each organ such as; the heart, liver, spleen, kidney, brain, forestomach, glandular stomach, and intestine. The tissues of these organs were used to extract the viral (DAdV-3) DNA and the viral load in each organ was determined by RT-PCR. The recombinant plasmid (pMD18-T-52k) was constructed with the target gene (52K). There were 1.18 × 10^10^ copies/µL, and the standard curve was y = −3.4955lg^X^ + 40.53(R^2^ = 1.0). The dissolution curves of standard samples showed a single peak without primer-dimer and a nonspecific peak. The threshold cycle (CT) value of each infected tissue and organ was obtained by amplification. DAdV-3 (AH-HF-2020) strain was found in all collected organs and tissues with different viral loads. The viral load in the liver was the highest, which was 8.3 × 10^6^ copies/g, followed by the spleen, duodenum, kidney, and glandular stomach, were 7.2 × 10^5^ copies/g, 6.9 × 10^5^ copies/g, 6.5 × 10^4^ copies/g, and 3.3 × 10^4^ copies/g, respectively, whereas the lowest viral load in the heart (1.9 × 10^4^ copies/g), muscle (1.7 × 10^4^ copies/g), muscular stomach (1.7 × 10^4^ copies/g), and brain (1.6 × 10^4^ copies/g). We found that the distribution of viral load was significantly different (*P* < 0.01) in each organ of infected birds (Figure 5).

### Cellular Changes Caused by DAdV-3 (HF-AH-2020) Infection

**Figure 6:**
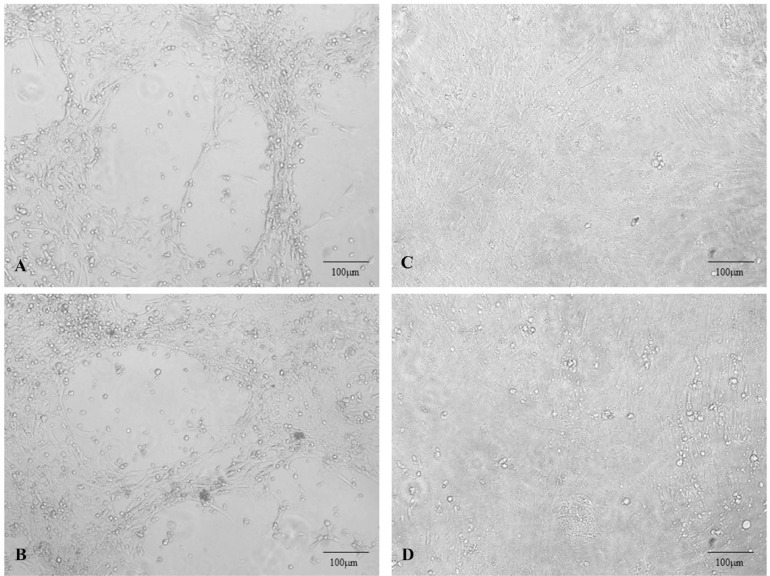
Cytopathic effects of DAdV-3 (HF-AH-2020) on CEK cells can see clearly; the morphology of infected CEK cells was changed from normal to round and clusters like grapes. (A) CEK cells Infected by 30 µL grinded liver solution of infected Muscovy duck. (B) CEK cells infected via allantoic fluid of infected chicken embryo. (C) and (D) CEK cells infected with PBS (negative control). The pictures were taken at 72 h after inoculation by using the scale bar of 100 μm.

CEK cells were used to identify changes in DAdV-3 infection. CEK cells were infected with the supernatant with 10 μL grinded liver tissues and 30 μL of allantoic fluid to observe the pathological changes (shrinking, clustering, and death) in CEK cells which were started from d 4 and gradually increased up to d 7. The infected cells showed poor refractive index, turned round, and became clustered like grapes, while on the other hand, the control negative cells remained normal (Figure 6).

### The TCID_50_ of the DAdV-3 (HF-AH-2020)

DAdV-3 caused cytopathic effect (**CPE**) in LMH cells during in vitro culture in 96-well plates. The cultured DAdV-3 was diluted 10 times and then used to infect the LMH cells in a 96-well cell culture plate to observe the cell changes. After 9 d of culture, the TCID_50_ of the virus calculated according to the Reed-Muench method was found to be 10^−3.24^/0.1 mL.

### Isolation of Bacteria and Identification of Virulence Island

Isolation and identification of bacteria from Muscovy duck liver were done by Gram staining and bacterial 16sRNA. The results of Gram staining and bacterial 16sRNA showed that the isolated bacteria was *Escherichia coli* with 5 virulence island genes including *Tsh* (*temperature-sensitive hemagglutinin*), *Cav* (*structural genes of colicin V operon*), *lss* (*increased serum survival*), *Irp2* (*iron-repressible protein*), and *lucd* (*aerobactin synthesis*).

### Necropsy Findings and Level of Antibodies

On the 7th and 14th day of age, all birds (chickens and ducks) were infected with DAdV-3 (HF-AH-2020) for 3 consecutive days (0.25 mL/d). No mortality was observed within 12 d of postinfection (Table 2). On fourth day after the infection, there were no obvious gross pathological and histopathological changes in livers and kidneys. The cloacal swab and liver samples were collected to detect the DNA of HF-AH-2020.

**Table 2:** Test results of infected SPF chicken and Muscovy duck.

Age of Infection	Groups/no. of birds	Dose, number, and days of infection	Number of mortality
7 days SPF chicken	Infected/15	0.25 mL/d for 3 consecutive days	0
	Control/15	0.25 mL PBS/d for 3 consecutive days	0
14 days SPF chicken	Infected/15	0.25 mL/d for 3 consecutive days	0
	Control/15	0.25 mL PBS/d for 3 consecutive days	0
7 days Muscovy ducks	Infected/15	0.25 mL/d for 3 consecutive days	0
	Control/15	0.25 mL PBS/d for 3 consecutive days	0
14 days Muscovy ducks	Infected/15	0.25 mL/d for 3 consecutive days	0
	Control/15	0.25 mL PBS/d for 3 consecutive days	0

On the 12th day of postinfection, 0.5 mL blood was drawn from the wing veins of both birds (chickens and ducks) via a syringe and serum was separated for the detection of antibody titers. When the experimental chicken and duck serum dilution reached 1:20,000, the OD450 nm value ranged from 2.32 to 2.93, while the control group ranged from 0.13 to 0.2, and there was no significant difference between chickens and duck antibody titers. Notably, the serum DAdV-3 antibody levels were higher in the infected birds than in the control group, with a positive rate of 100%.

### DAdV-3 (HF-AH-2020) Induced IFN-β and IFN-γ Production in Chicken Embryonic Kidney Cells

**Figure 7:**
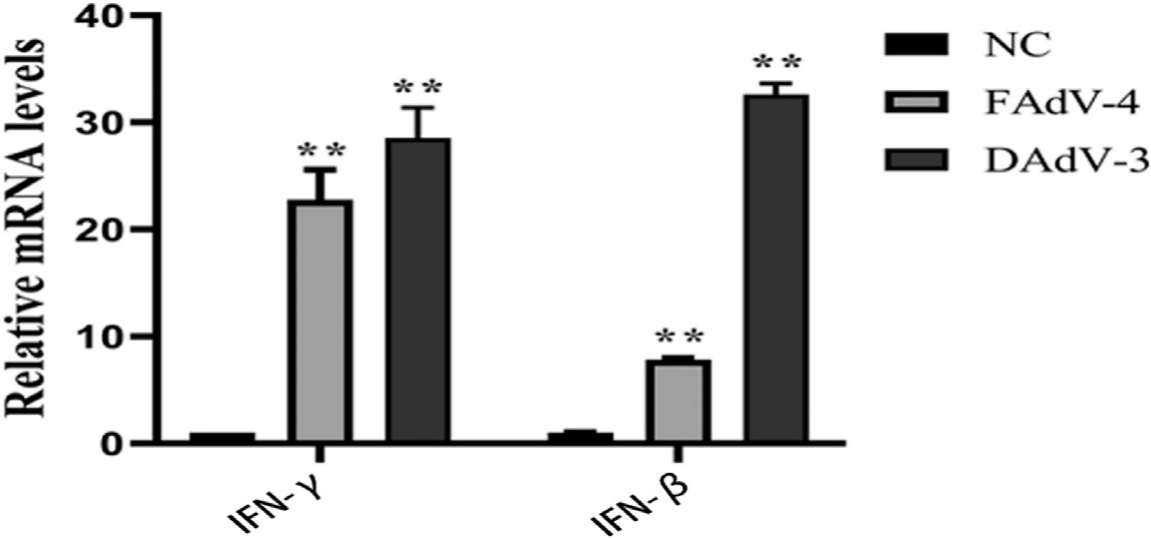
The expression levels of IFN-β and IFN-γ genes in CEK cells infected with DAdV-3 (HF-AN-2020) were significantly higher than the FAdV-4. Seventy-two hours of postinfected CEK cells were collected for detecting the expression of IFN-β and IFN-γ genes using qRT-PCR analysis. ∗∗ Indicate the highly significant difference (*P* < 0.01).

IFN-β and IFN-γ are immunomodulatory and antiviral cytokines that play an important role in host antiviral response. To understand the transcription levels of interferon in infected cells, IFN-β and IFN-γ expression levels were determined by qRT-PCR. The expression level of the IFN-β and IFN-γ genes in DAdV-3-infected cells was significantly higher than the FAdV-4 infected group 3 days postinfection (Figure 7). The gene expression of *IFN-β* in FAdV-4 and DAdV-3 infection was increased by 7.9 and 32.6 times respectively while the expression level of IFN-γ in FAdV-4 and DAdV-3 was increased by 22.8 and 28.6 times, respectively. The statistical analysis showed that the gene expression of IFN-β and IFN-γ was significantly different from the control group (*P* < 0.01), indicating that the ability of DAdV-3 to induce interferon was stronger than FAdV-4.

### Cellular Pathway of IFN-β and IFN-γ Production

**Figure 8:**
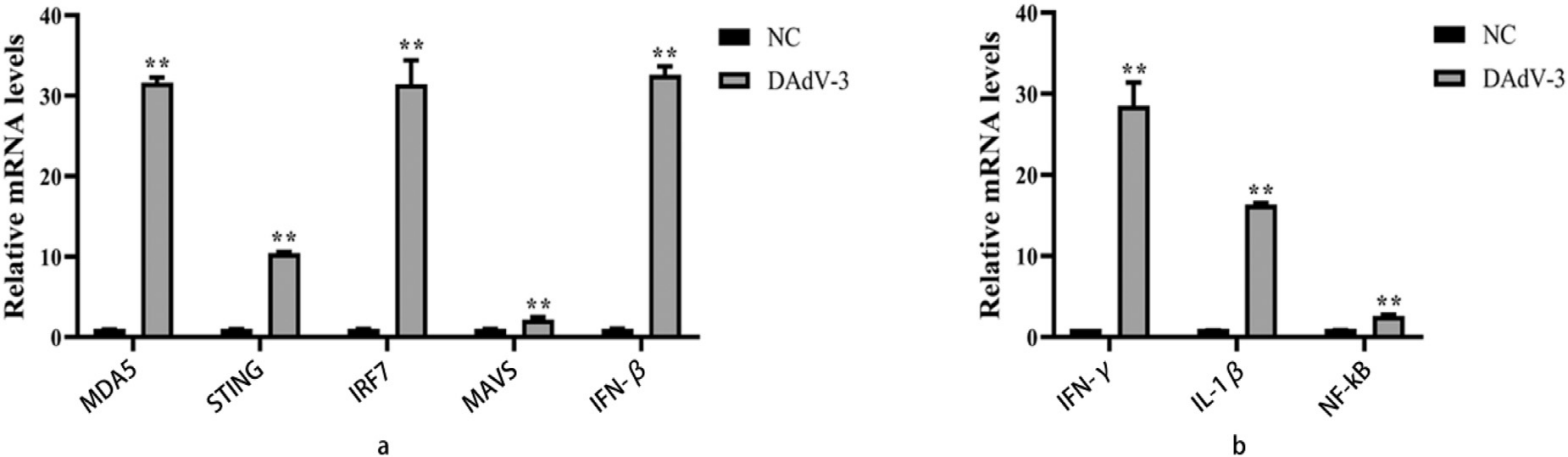
The expression levels of STING, NF-κB, and their related genes in CEKs infected with DAdV-3 (HF-AN-2020) were observed, and 72 h of postinfected CEK cells were collected for the detection of MDA5, STING, IRF7, MAVS, IFN-β, NF-κB, IFN-γ, and NF-κB genes using qRT-PCR. (A); STING pathway (B); NF-κB pathway ∗∗ indicate the highly significant difference (*P* < 0.01).

To explore the signaling pathway, the DAdV-3 stimulates the cells to produce interferons identified in this study. We verified that the expression level of MDA5, STING, IRF7, and MAVS rises to 31.6, 10.5, 31.4, and 2.2 times, respectively, compared to the control group (*P* < 0.01) (Figure 8A). The virus-stimulated CEK cells significantly increased the expression level of *NF-κB* and *IL-1β* up to 2.6 and 16.3 times respectively than the control group (*P* < 0.01) (Figure 8B). The result suggested that DAdV-3 could induce *IFN-β* and *IFN-γ* gene expression in the cells to enhance the immune response by STING and NF-kB pathways.

## DISCUSSION

According to the ICTV Taxonomy released in 2020, Duck adenovirus (**DAdVs**) includes 2 genera of duck *adenovirus*-A (**DAdV-A**) and duck *adenovirus*-B (**DAdV-B**). DAdV-A (DAdV-1) is also known as egg drop syndrome virus (**EDSV**) (Kang et al., 2017). DAdV-B is also known as Duck *adenovirus* type 2 (**DAdV-2**). The phylogenetic analysis of the *hexon* gene suggests that the DAdV-3 also belongs to DAdV-B, but DAdV-4 isolated from China in 2020 which is an independent branch between FAdV and DAdV-B (Huang et al., 2020). The full-length amino acid homology of the isolated DAdV-3 (HF-AH-2020) strain with the reported DAdV-3 (KR135164) strain was 100%, while with DAdV-2, DAdV-4, and goose adenovirus 4 (**GoAdV-4**) was 92.3, 57.2, and 57.4%, respectively (Table 1).

There were 4 amino acid differences (ORF19B, ORF66, and ORF67) in the genome of HF-AH-2020 and GD-CH-2014 strains. The early termination of ORF67 in the HF-AH-2020 strain was the same as reported elsewhere (Shi et al., 2022). Whether these amino acid differences lead to changes in virulence remains to be further investigated. The homology of DAdV-3 main structural proteins (hexon, fiber-1, and fiber-2) is higher than that of DAdV-2 and DAdV-4. Many researchers have found that the fiber-2 protein of both FAdV and DAdV can stimulate the host's body to produce antibodies, which is an ideal antigenic site for vaccine development (Yin et al., 2019; Tian et al., 2020). It indicates that the vaccine prepared by fiber-2 of DAdV-3 may also have efficacy in protecting the DAdV-2 infection. Fiber-1 mediates the virus adsorption for FAdV-4 (Zou et al., 2021). Previous studies have found that the different serotypes of *Adenoviruses* are prone to recombination, especially FAdV-D and FAdV-E (Schachner et al., 2019). The DAdV-2, DAdV-3, and DAdV-4 are isolated from Muscovy ducks like anseriform birds, DAdV-2 and DAdV-3 belong to the same branch as GoAdV-4 and might have come from the recombination of DAdV-2 and GoAdV-4, and DAdV-4 may be the product of the recombination of FAdV and DAdV-B. There is a recombination between different serotypes of adenovirus. The viral load in chicken embryos showed that the virus can infect multiple organs, mainly the liver, spleen, intestine, and kidney. The distribution of viral load in different body organs was similar to that of other serotypes of DAdVs (Wang et al., 2015; Shao et al., 2019a,b).

Muscovy duck is an important host of DAdVs. The pathogenicity of DAdV-3 (HF-AH-2020) was very low which can cause only muscle fatigue in Muscovy ducks but it also induces a high level of cellular immunity. While typical organ changes were found during the autopsy. At the same time, the pathological sectioning of the liver and kidney of the infected animals was performed, but no pathological changes were observed. To determine the virulence level of allantoic fluid and grinded liver solution of infected Muscovy duck was inoculated on CEK cells. The results showed that both allantoic fluid and infected grinded liver solution have the same virulence and the same cytopathic time for CEK cells. In our previous study, we found that FAdV-4 can induce IFN expression through the NF-κB pathway. However, CEK cells without DAdV-3 (HF-AH-2020) inoculation could not produce IFN.

The DAdV-3 weakened strain (HF-AH-2020) can induce high-level expression of IFN-β and IFN-γ. Our results indicate that the expression of IFN-β and IFN- γ increased 32.6 and 28.6 times, respectively, after DAdV-3 (HF-AH-2020) infection with CEK cells, this high expression level of IFN-β and IFN-γ plays an important role against virus infection. Furthermore, we found that the DAdV-3 (HF-AH-2020) induces the IFN-β by the STING pathway, and the expression levels of related genes *MDA5, STING, IRF7*, and *MAVS* were 31.6, 10.5, 31.4, and 2.2 times respectively higher than the control group. The expression of *IFN-γ* and related pathways such as NF-κB and IL-1β increased 2.6 and 16.3 times respectively. The statistical analysis of correlation showed that the difference in expression was significant (*P* < 0.01) after CEK infection with DAdV-3, and the expression of *IF-1β* increased 16.3-fold. This shows that DAdV-3 is similar to FAdV-4 and can be induced by STING and NF-κB pathways. This indicates that STING receives the pathogen recognition signal transmitted by MDA5 through MAVS, and then activates IFN-β through downstream IRF7 to play an antiviral role. Meanwhile, the HF-AH-2020 can activate the NF-κB pathway to activate the expression of IFN-γ and IL-1β. Therefore, this strain (HF-AH-2020) is different from the other isolates of other scholars and is a good attenuated vaccine.

At present, the incidence of DAdV-3 in the clinic is getting higher and higher, and even appears to infect other birds (such as chickens and geese), which should be paid special attention to. This infection characteristic is similar to other adenoviruses, for example, the DAdV-1 from Muscovy ducks has no evidence of a pathogenic effect in ducks but it can infect chickens by affecting their egg quality and production. In addition, *Adenovirus* is also prone to infection with bacteria or immunosuppressive hosts with an increased mortality rate (Jiang et al., 2019; Yu et al., 2019).

## CONCLUSIONS

In this study, a strain HF-AH-2020 of DAdV-3 was isolated and identified, which showed broad tissue tropism in multiple organs via in-vivo, with the highest content in the liver and the lowest content in the brain. The DAdV-3 (HF-AH-2020) infects the chickens and Muscovy ducks without mortality. DAdV-3 (HF-AH-2020) produces INF-β and IFN-γ in CEK cells through STING and NF-κB pathways, respectively. Genomic analysis showed that there were 4 amino acid differences between low (HF-AH-2020) and high (GH-CH-2014) pathogenic strains, which were located at ORF19B, ORF66, and ORF67, which indicate mutation, and that mutation play an important role in the pathogenicity of DAdV-3 (HF-AH-2020).
